# When to resume bariatric surgery after COVID-19 pandemic?: results of patients’ and surgeons’ survey

**DOI:** 10.1186/s12893-021-01145-y

**Published:** 2021-03-15

**Authors:** Alicja Dudek, Michał Wysocki, Maciej Walędziak, Jacek Szeliga, Monika Proczko-Stepaniak, Michał Pędziwiatr, Piotr Major

**Affiliations:** 1 2ndDepartment of General Surgery, Jagiellonian University Medical College, Jakubowskiego 2 st., 30-688 Krakow, Poland; 2 Department of General, Oncological, Metabolic and Thoracic Surgery, Military Institute of Medicine, Warsaw, Poland; 3 Department of General, Gastroenterological, and Oncological Surgery, Collegium Medicum Nicolaus Copernicus University, Torun, Poland; 4 Department of General, Endocrine and Transplant Surgery, Medical University of Gdansk, Gdansk, Poland

**Keywords:** Bariatric care after the pandemic, Bariatric surgery, SARS-CoV-2 pandemic

## Abstract

**Background:**

Coronavirus Disease 2019 is affecting most countries around the world, including Poland. In response, all elective surgeries have been postponed. We asked patients and surgeons when they want bariatric surgery to resume after pandemic. The main aim of the study was to determine patients’ and surgeons’ expectations about when to resume bariatric surgery regarding COVID-19 pandemic state.

**Methods:**

The study was conducted in two groups: Group 1—bariatric patients; Group 2—bariatric surgeons. Two online surveys were distributed.

**Results:**

A total of 895 patients, 299 before, 596 after surgery and 32 surgeons took part in survey. All patients and surgeons declared willingness to resume bariatric surgeries after pandemic and responded that they should be resumed immediately the World Health Organization announces end of pandemic (42%). The majority of patients before surgery answered that bariatric procedures should be resumed immediately the number of daily incidents begins to decrease (53%). In the patient group, current body mass index (p < 0.001) and contact with COVID+/quarantined persons (p < 0.001) had impact on the response to resumption of bariatric procedures.

**Conclusions:**

Patients opted to wait for bariatric surgery until the oncological queue has become shorter. Surgeons presented a readiness to resume both procedures in parallel.

## Background

The COVID-19 is affecting most countries around the world, including Poland [1]. The coronavirus pandemic has had a radical impact on the functioning of healthcare systems worldwide. The health crisis has also brought new challenges to surgical care, including bariatric surgery. In response to the COVID-19 pandemic, a strategy of postponing elective surgery has been adopted by most surgical societies [2, 3]. Bariatric surgery is one of the first disciplines that has largely implemented this strategy. According to *The International Federation for the Surgery of Obesity and Metabolic Disorders* (IFSO) recommendations, all elective surgical and endoscopic cases for metabolic and bariatric surgery should be postponed in the interim. Furthermore, clinic and hospital visits are not recommended [4], as other surgical and non-urgent oncological procedures have also been recommended to be cancelled. However, promises to resume procedures at the earliest available date once the pandemic subsides have been offered by the healthcare authorities. The extended waiting lists at surgeries will be another important issue after the pandemic.

The number of bariatric procedures performed before the pandemic was insufficient in relation to the rising prevalence of obesity and the needs of bariatric patients worldwide [5, 6]. In addition, the waiting list for bariatric procedures is growing rapidly as a result of the pandemic. The increasing negative effects of a pandemic outbreak affect bariatric surgeons, surgical centers, patients and health care systems. Current efforts are mainly focused on reorganizing the functioning of hospitals and wards to defeat the coronavirus. There is a lack of data on plans for post-pandemic bariatric care organizations.

To effectively prepare bariatric teams, bariatric care organizations should implement their strategies immediately after the pandemic has ended. An appropriate timing for the resumption of bariatric procedures should be determined first.

## Methods

The aim of the study was to define an appropriate time to resume bariatric surgery after the end of the SARS-COV-2 pandemic.

### Study design

The study was conducted between 8 and 22 April 2020 in two groups: Group 1—bariatric patients, Group 2—bariatric surgeons. Two anonymous online surveys were designed. Surgeons from four bariatric centers were responsible for distribution of survey and gathering of material. The first survey was prepared for patients from the Polish *Association of Bariatric Patients* (CHLO) and distributed online among patients. The data collected in the patient survey were also analysed on the impact of the pandemic on long-term effects of bariatric treatment [7]. The second survey was sent to all bariatric surgeons associated with the *Metabolic and Bariatric Chapter at the Association of Polish Surgeons* (SCMiB) who were invited to take part in the study by recruiting surgeons via e-mail through the SCMiB mailing list. In order to disseminate the survey to surgeons and encourage as many participants as possible, the survey also appeared on the society's official website. The invitation to the project and the introduction to the questionnaire give details of the study design, assumptions of the study and types of questions. The survey included precise guidelines on how to be fulfilled. Participation in both surveys was voluntary. Participants were required to give their consent to participate in the study as their response to the first survey question, which also supplied information on how to withdraw consent.

### Inclusion and exclusion criteria

Group 1 included patients who were members of CHLO and who answered all the questions in the survey. The group contained patients before and after surgery. Cancer patients or patients with complications after bariatric interventions whose surgery could not be postponed were advised not to complete the questionnaire. Group 2 included surgeons and general surgery residents who are members of SCMiB working in bariatric centers. All eager participants in the association, regardless of certification, were included in the study. However, inactive surgeons (due to retirement, sick leave, vacation, or other reason for being out of practice during the pandemic) were excluded.

### Survey

Questionnaires for both study groups were accessible online. There was no time limitation on completion. Both surveys were divided into two parts.

The first part consisted of questions covering consent to participate and basic demographic data characterizing the groups of patients and surgeons. The group of patients was asked to state their age, sex, current body mass index (BMI), obesity comorbidites, whether they had undergone bariatric procedures in the past, contact with COVID+/quarantined persons and COVID status of the hospital taking care of them. Questions to the surgeon group concerned age, sex, stage of surgical training, type of employing hospital, COVID status of employing hospital and number of bariatric procedures performed in 2019 and 2020 until the start of the pandemic.

**Fig. 1:**
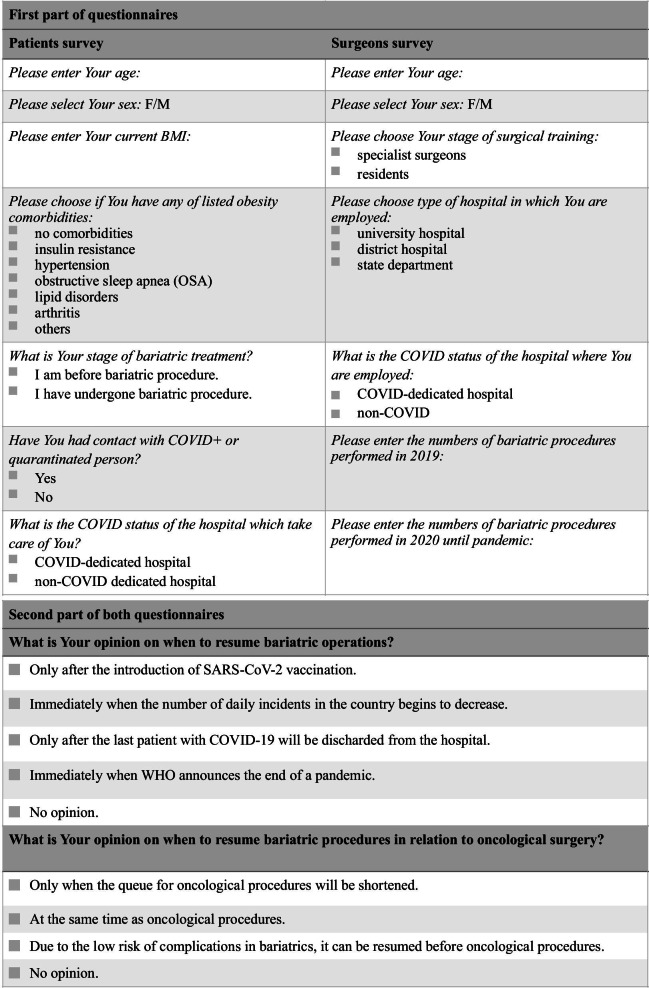
Questionsasked in surveys—patients and surgeons group

The second part included the same questions in both surveys. A group of patients and a group of surgeons were first asked their opinion on when bariatric surgery should be reinstated. In view of the lengthening waiting list for other elective procedures, especially oncological operations, both surveys then asked for the respondent’s opinion on how to reconcile the waiting lists for bariatric procedures with those for other elective surgery, including oncological procedures (Fig. 1).

### Statistical analysis

Results were presented as mean plus standard deviation or median with interquartile range. To assess factors that could potentially influence the responses provided, univariate and multivariate regression models were run on Statistica 13.5 PL (Statsoft, USA).

## Results

A total of 927 participants took part in the survey (895 patients/32 surgeons). The study group included 792 (85%) females and 135 (15%) males. The median age was 39 (33–45). Respondents were associated with both COVID-dedicated hospitals (22%) and non-COVID-dedicated hospitals (77%).

### Group 1 characteristics (patients)

The median age of the patient group was 39 (33–45). The study group included 786 females (88%) and 109 males (12%). The median current BMI was 34.77 (29.3–41.18). 74% of patients presented with obesity comorbidities. The patients who participated in the study were from both COVID-dedicated hospitals (198; 22.1%) and non-COVID-dedicated hospitals (697; 77.9%). 56 of the patients (6.26%) had had contact with COVID+ or a person who was quarantined. The patient study group was divided into two subgroups: patients before surgery and patients and after surgery (299/596). Basic characteristics of both subgroups are presented in Table 1.

**Table 1:** Basic characteristics of group 1

	All patients	Patients before surgery	Patients after surgery
n (%)	895 (100%)	299 (33%)	596 (67%)
Median age, years (IQR)	39 (33–45)	38 (32–44)	39 (33–45)
Males/Females, n (%)	109/786 (12%/88%)	39/260 (13%/87%)	69/527 (12%/88%)
Median BMI, kg/m^2^ (IQR)	34.77 (29.3–41.18)	42.61 (38.99–47.63)	31.21 (27.54–47.63)
Obesity comorbidities, n (%)	661 (74%)	239 (80%)	422 (71%)
Contact with COVID+/quarantined person	56 (6.26%)	10 (3.34%)	46 (7.72%)
Hospital of patient care			
COVID-dedicated hospital	198 (22.12%)	79 (26.42%)	119 (19.97%)
Non-COVID dedicated hospital	697 (77.88%)	220 (73.58%)	477 (80.03%)

### Group 2 characteristics (surgeons)

The mean age of surgeons who participated the survey was 42 ± 10. The group contained 26 males (81%) and 6 females (19%). The group contained 26 specialist surgeons (81%) and 6 residents (19%). 53% of the respondents were employed in university hospitals, 38% of them in district hospitals and 9% in state departments. 12 surgeons were working in the COVID-dedicated hospitals (38%), while 20 in non-COVID-dedicated hospitals (63%). Median number of bariatric surgeries provided altogether in the hospital in 2019 was 150 (82–200). Median number of bariatric surgeries performed overall in 2020 until the start of the pandemic was 29 (20–50) (Table 2).

**Table 2:** Basic characteristics of group 2

	Bariatric surgeons
n (%)	32 (100%)
Mean age, years ± SD	42 ± 10
Males/females, n (%)	26/6 (81%/19%)
Stage of surgical training	
Specialist surgeons, n (%)	26 (81%)
Residents, n (%)	6 (19%)
Type of employing hospital	
University hospital, n (%)	17 (53%)
State department, n (%)	3 (9%)
District hospital/city hospital, n (%)	12 (38%)
COVID status	
COVID-dedicated hospital	12 (37.5%)
Non-COVID dedicated hospital	20 (62.5%)
Numbers of bariatric procedures	
Median number of bariatric procedures in 2019, n (IQR)	150 (82–200)
Median number of bariatric procedures in 2020 until pandemic, n (IQR)	29 (20–50)

### When bariatric procedures should be resumed

**Fig. 2:**
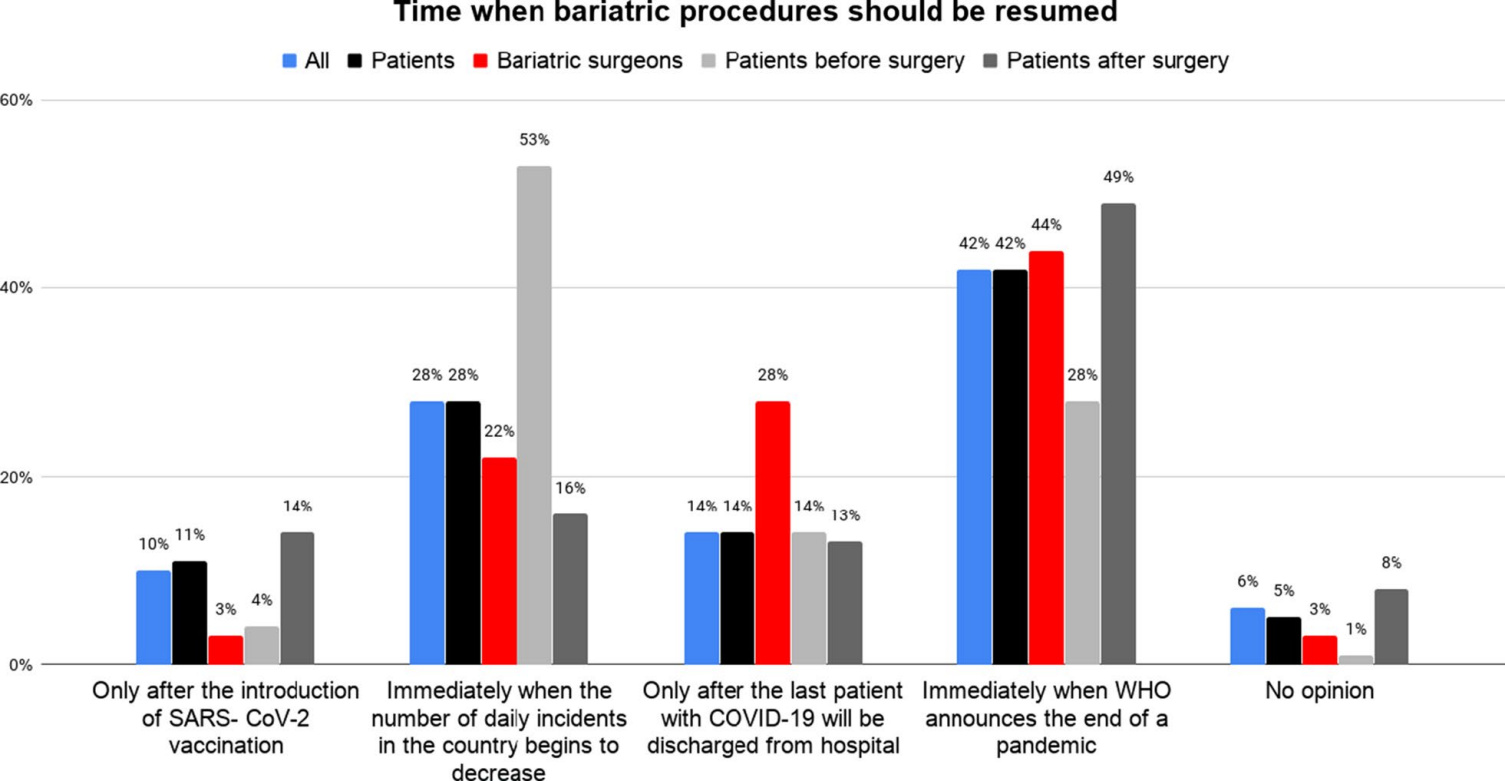
Time when bariatric procedures should be resumed

All patients and surgeons declared a high willingness to resume bariatric surgery after the SARS-CoV-2 pandemic, with a median score of 10 (10–10). There was no statistically significant difference between patients and surgeons in their answers to questions on the timing for resuming bariatric procedures. A majority in both groups supported the position that bariatric surgery should be resumed as soon as the World Health Organization (WHO) declares an end to the pandemic**:** 377 (42.4%), 14 (43.8%). The distribution of the remaining answers was as follows: immediately the number of daily incidents in the country begins to decrease: 251 (28.1%), 7 (21.9%); only after the last patient with COVID-19 has been discharged from hospital: 121 (13.5%), 9 (28.1%); only after the introduction of SARS- CoV-2 vaccinations: 95 (10.6%), 1 (3.1%); no opinion: 51 (5.4%), 1 (3.1%) (Fig. 2).

Answers given by the two subgroups of patients (before and after surgery) presented two statistically-significant different approaches to the question asked. The majority of patients in the before surgery subgroup answered that bariatric procedures should be resumed immediately the number of daily incidents in the country begins to decrease**:** 157 (52.5%), while the most popular answer in the after surgery subgroup was immediately WHO announces the end of the pandemic: 291 (48.8%).

Being associated with a COVID-dedicated hospital might have had an impact on the opinions of both groups on restoring bariatric surgery: after WHO announces the end of the pandemic (p = 0.02) and only after the last patient with COVID-19 has been discharged (p < 0.001).

Among the patient group, current BMI (p < 0.001) and contact with a COVID+/quarantined person (p < 0.001) influenced opinions on resumption of bariatric procedures after daily incidents in the country start to decrease. Answering that resumption should take place immediately WHO announces the end of the pandemic might be related to the status of surgery for that patient (p < 0.001). The statistical analysis indicated that factors like stage of surgical training, type of employing hospital, COVID status of employing hospital and number of bariatric procedures in 2019 or in 2020 until the start of the pandemic did not influenced the opinions given by surgeons (p > 0.050).

### Resumption of bariatric procedures in relation to oncological surgery

**Fig. 3:**
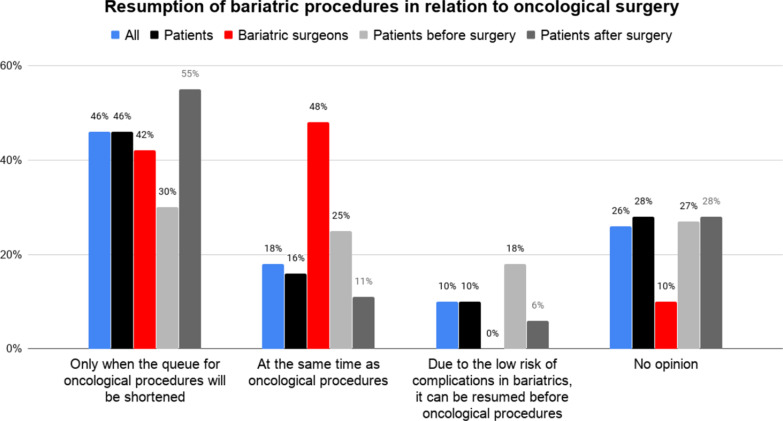
Resumption of bariatric procedures in relation to oncological surgery

The answers of the patient and surgeon groups to the question when bariatric procedures should be resumed in relation to oncological surgery differed significantly. The majority of patients opted for resumption of bariatric procedures only after managing the oncological queue: 396 (46%). However, most surgeons stated that bariatric procedures should be resumed in parallel with oncological procedures: 15 (48.4%). Analyzing the two subgroups, both in patients before and after surgery, the majority pointed out that shortening the queue for oncological procedures should be the priority: 90 (30.1%), 306 (54.5%). However, there was a significant difference in opinions on the parallel resumption of oncological and bariatric procedures between patients before and after surgery: (74 (25%) vs. 67 (11.4%) p < 0.001) (Fig. 3).

## Discussion

The study presented here is the result of a national survey, the aim of which was to begin discussion with both surgeons and patients on the most appropriate time to resume bariatric surgery. The study showed a high willingness by all participants to resume bariatric surgery immediately after the SARS-CoV-2 pandemic ends. The survey affirmed high patient expectations for bariatric operations to be resumed as soon as WHO announces the end of the pandemic.

The value of the study is to highlight patients' perspectives on the timing of resumption of bariatric surgery suspended due to the prevailing SARS-CoV-2 pandemic, as well as patients' readiness for surgery, which could be affected by decreased motivation, difficult preparation for surgery, or concerns about hospitalization in the era of the pandemic.

To broadly analyze the impact of the pandemic on bariatric care in Poland, we conducted a survey with bariatric surgery specialists, who, through a survey, were asked about the feasibility of bariatric care during the pandemic, their bariatric center status (COVID/non COVID), and asked their views on the impact of the pandemic on the future of bariatric care [8]. The results of the study led to the conclusion that bariatric surgery specialists are ready to resume surgery as soon as the pandemic subsides and that they do not believe the pandemic will affect the safety of bariatric surgery. The results prompted us to consider bariatric patients' views compared to specialists on when to resume bariatric surgery and to discuss issues that may influence their decisions.

According to the latest data, obesity may present a higher risk for severe illness from COVID-19 and it can significantly worsen the prognosis of pneumonia [9, 10]. Sockalingam et al. point out the tremendous psychological impact of the pandemic; an increase in eating psychopathology and binge eating syndrome can have a strong negative effect on obesity management and gaining weight [11]. The group of patients who experienced delays in the preoperative period showed a greater desire to resume surgery as soon as the pandemic curve flattens compared to patients after surgery. The dominant opinion among patients who had completed their treatment was to wait until WHO announces the end of the pandemic. Current BMI and contact with a COVID+/quarantined person should be taken into consideration as the most likely factors to influence patient opinions. Those findings may be related to patients who fear that they are in the high-risk group feeling a desperate need for surgery. On the other hand, many scientists are warning of the likelihood of a second wave of the epidemic in the fall [12]. The prospect of a rebound of the epidemic may also be increasing patients’ desperation for surgery. The surgeon group showed a high readiness to provide bariatric surgery treatment as soon as possible after the end of the pandemic. The COVID status of the employing hospital does not seem to have had an influence on the answers given.

The effect of bariatric treatments is not only weight loss, but also a reduction in the number of coexisting diseases, and further improving the quality of life. A good example of how important bariatric treatments play a role in improving the quality of life is the problem of Obstructive Sleep Apnea (OSA). Bariatric surgeries reduce the symptoms of OSA and improve sleep quality and daytime sleepiness [13]. Lack of possibility of surgical treatment results in shortened quality adjusted life years.

What is more, the lack of an appropriate quality of sleep lowers immunity, which may additionally cause patients' anxiety associated with pandemic situation and, on the other hand, may increase their desire to take up surgical treatment quickly [14].

Another challenge in the case of restoring bariatric procedures will be the need to observe whether the SARS-CoV-2 infection affects the longterm effects of surgical treatment. Similar studies have so far been designed for other types of infections [15].

In the context of oncological treatment, patients opted to wait for bariatric surgery until the oncological queue is shorter. However, surgeons showed a readiness to resume both procedures in parallel. A prioritization strategy should be established that is appropriate to the readiness of surgeons and the needs of patients.

This study is associated with several limitations. Firstly, possible limitations of our study include recall bias and the subjectivity of patients and surgeons in stating their opinions. Secondly, free text comments provided by participants were not collected in the survey. It might be worthwhile extending the study to include a qualitative analysis. Additionally, the survey was limited to patients who are members of the Polish *Association of Bariatric Patients* (CHLO) and may not reflect the entire bariatric population; however, it is worth noting that CHLO members include a significant proportion of bariatric patients from all over the country. The second survey was dedicated only to bariatric surgeons. The group of surgeons specializing in bariatrics in Poland is limited. Asking for the opinion of all surgeons could be valuable. As a final point, it should be noted that we concentrated only on Polish bariatric departments and Polish patients so it may be difficult to extrapolate our results to other countries; however, we anticipate that most bariatric surgeons and patients have similar problems.

## Conclusions

A timing for the resumption of bariatric procedures should be established in line with the needs of patients and the readiness of surgeons. The overall opinion of both patients and surgeons was to resume bariatric procedures immediately WHO announces the end of the pandemic. Patients before surgery preferred to resume bariatric procedures immediately the number of daily incidents in the country begins to decrease. Patients prefered to wait for bariatric surgery until the oncological queue has become shorter. Surgeons demonstrated a willingness to resume bariatric procedures in parallel with oncological surgery immediately after the pandemic ends.

## Data Availability

The datasets generated during and/or analysed during the current study are available in the Jagiellonian University Medical College repository. The data are hosted on a server operated by the Jagiellonian University Medical College. Data are available from the authors upon reasonable request and with permission of Jagiellonian University Medical College.

## Abbreviations

WHO: World Health Organization
IFSO: The International Federation for the Surgery of Obesity and Metabolic Disorders
CHLO: Association of Bariatric Patients
SCMiB: Metabolic and Bariatric Chapter at the Association of Polish Surgeons
BMI: Body mass index
